# Intratumoral STING Agonist Injection Combined with Irreversible Electroporation Delays Tumor Growth in a Model of Hepatocarcinoma

**DOI:** 10.1155/2021/8852233

**Published:** 2021-01-27

**Authors:** Aritz Lasarte-Cia, Teresa Lozano, David Cano, Celia Martín-Otal, Flor Navarro, Marta Gorraiz, Noelia Casares, Isabel Vivas, Juan José Lasarte

**Affiliations:** ^1^Immunology and Immunotherapy Program, Center for Applied Medical Research (CIMA), University of Navarra, 31008 IDISNA, Pamplona, Spain; ^2^Department of Radiology, Clínica Universidad de Navarra, Pamplona, Spain

## Abstract

**Background/Aim:**

Irreversible electroporation (IRE) showed promising results for small-size tumors and very early cancers. However, further development is needed to evolve this procedure into a more efficient ablation technique for long-term control of tumor growth. In this work, we show that it is possible to increase the antitumor efficiency of IRE by simmultaneously injecting c-di-GMP, a STING agonist, intratumorally.

**Materials and Methods:**

Intratumoral administration of c-di-GMP simultaneously to IRE was evaluated in murine models of melanona (B16.OVA) and hepatocellular carcinoma (PM299L).

**Results:**

The combined therapy increased the number of tumor-infiltrating IFN-*γ*/TNF-*α*-producing CD4 and CD8 T cells and delayed tumor growth, as compared to the effect observed in groups treated with c-di-GMP or IRE alone.

**Conclusion:**

These results can lead to the development of a new therapeutic strategy for the treatment of cancer patients refractory to other therapies.

## 1. Introduction

Irreversible electroporation (IRE) is an emerging alternative to multimodal ablative therapies for the liver [1], prostate [2], kidney [3], pancreas [4–6], or lung cancers [7]. The main use of IRE is aimed at the ablation of tumors that are in contact with vital vascular or nervous structures which must be preserved. Electroporation destroys tumor cells but it does not affect collagen-containing structures like vessels and nerves [8–10]. The advantages of IRE compared to other techniques are as follows: (i) the selectivity of the tissue affected [10]; (ii) the ability to specifically define the margins affected by the procedure [11]; (iii) the short time the treatment lasts; and (iv) the possibility of monitoring the effect of electroporation in real time [11]. All this makes IRE a therapeutic alternative in patients with tumors located in areas not surgically resectable near to vital structures.

Clinical trials showed safety and absence of serious adverse effects when IRE was used; however, its therapeutic efficacy remained poor [5, 12, 13]. It was suggested that there are islands of viable tumor cells remaining within ablated regions after IRE treatment, which may contribute to tumor development [14]. Lack of long-term efficacy of this technique might also be due to its limited capacity to induce an inflammatory reaction that favors the activation of an antitumor immune response. This is because IRE causes tumor cell death by apoptosis and not necrosis as in other techniques based on thermal ablation or radiation [15]. In previous work, we found that it was possible to improve the antitumor effect of IRE when combining it with the intratumoral injection of Poly-ICLC (Hiltonol) immediately before the IRE procedure [16]. Poly-ICLC is a synthetic analog that mimics double-stranded viral RNA, a ligand of pattern recognition receptors (PRR) including TLR3, MDA5, RIG-1, or the NLRP3 inflammasome that sense danger signals [17]. In addition to RNAs, double-stranded DNAs (dsDNA) are potent inducers of type I interferons (IFNs). There are a number of sensors of cytosolic dsDNA which can trigger different signaling pathways through the endoplasmic reticulum membrane protein STING (stimulator of IFN genes) [18] [19]. Indeed, in the presence of cytosolic double-stranded DNA (dsDNA), activated cyclic GMP-AMP synthase (cGAS) uses cytosolic ATP and GTP as substrates to catalyze the production of cyclic dinucleotides (CDNs) (reviewed in [20]). Upon binding to CDNs, STING translocates from the ER to the Golgi apparatus and further to the perinuclear microsomes and activate TBK-1/IRF-3 and NF-*κ*B signaling pathways inducing robust type I IFNs and proinflammatory cytokines, which can trigger adaptive immune responses against tumors [21, 22]. A number of natural and synthetic STING agonists are being tested in preclinical models and in the clinic for the immunotherapy of cancer. However, these molecules are susceptible to enzymatic degradation, having low bioavailability in target tissues and producing unwanted toxicities. New drug delivery systems are being explored to address these challenges [23].

Our main goal in the present work was to evaluate the effectiveness of IRE concomitant to the administration of a STING agonist to improve mice survival after a long-term follow-up. We have made the proof of concept in murine models of melanoma and hepatocarcinoma.

## 2. Materials and Methods

### 2.1. Cell Lines and Mice

B16-OVA (ATCC, American Type Culture Collection) and PM-299L (provided by Dr. Lujambio, NY) cell lines were cultured in RPMI-1640 supplemented with 10% FCS, 100 U/mL penicillin, 100 *μ*g/mL streptomycin, 2 mM L-glutamine, and 50 *μ*M 2-mercaptoethanol (CM medium). Specific pathogen-free, 7-10-week-old female C57BL/6 wild-type mice (Charles River) were used in agreement with the ethical directives of the Spanish veterinary authorities. They were housed in appropriate animal care facilities during the experiments and handled following the international guidelines required for experimentation with animals. Institutional ethical committee approved the experiments (Ref. 111-15).

### 2.2. In Vivo Experiments: Ire Treatment and Tumor Follow-Up

B16.OVA melanoma cells or PM299L HCC cells were injected (5x10^5^ cells/mouse), subcutaneously (s.c.) in C57BL/6 mice (*n* = 5 − 8) purchased by Harlan (Barcelona, Spain). Ten days after tumor cell injection, when the tumors grew to 5 mm in diameter, mice were randomly distributed into different experimental groups.

Irreversible electroporation was carried out using the ECM 830 Square Wave Electroporation System, using specific tweezers (edges of 2 mm) for the fixation of the tumor for the IRE treatment. IRE consisted in twenty consecutive pulses of 2500 V/cm (0.1 msec each) with 0.5 s intervals between pulses. When indicated, 25 *μ*L of a solution containing 1 mg/mL c-di-GMP STING agonist (InvivoGen) was injected intratumorally into the space defined by the tweezers. In an experimental group, c-di-GMP administration was done immediately before electroporation (IRE + c-di-GMP group). In another experimental group (c-di-GMP group), c-di-GMP was administered intratumorally exactly as described above, but without the administration of the electroporation current. IRE group received only the electroporation treatment alone without the c-di-GMP administration. Tumor size, represented as the multiplication of two perpendicular diameters (mm^2^), was measured at different time points. According to the institutional guidelines, mice were sacrificed if the mean tumor diameter was greater than 20 mm^2^.

### 2.3. Flow Cytometry

For characterization experiments, PM299L tumor-bearing mice were treated as indicated, and 10 days later, mice were sacrificed to analyze immune infiltrate by flow cytometry. Tumors were excised and digested with collagenase D (400 U/mL) and DNase-I (50 *μ*g/mL, Roche) for 20 min at 37°C. The spleens were mashed in PBS. Red blood cells were lysed by ACK buffer (Sigma). For functional analyses, cells were stimulated with PMA (50 ng/mL) and ionomycin (1 *μ*g/mL) in the presence of GolgiStop and GolgiPlug (BD Biosciences). After 5 hours, cells were incubated with Zombie NIR Fixable dye (BioLegend) and stained with fluorochrome-labeled mAbs against CD45.2 (104), CD8 (XMG1.4), CD4 (RMA4-5), and CD44 (IM7) in the presence of purified anti-CD16/32 mAb. For intracellular staining, cells were treated with the BD Fixation/Perm buffer (BD Biosciences) and stained with anti-IFN-*γ* (XMG1.2) and anti-TNF*α* (MP6-XT22) mAbs. Samples were acquired on a FACSCanto-II cytometer (BD Biosciences). Data were analyzed using the FlowJo software (TreeStar).

### 2.4. Statistical Analysis

Normality was assessed with the Shapiro-Wilk *W* test. Statistical analyses were performed using parametric (Student's *t* test and one-way ANOVA with Tukey's multiple comparison) and nonparametric (Mann–Whitney *U* and Kruskal-Wallis) tests. GraphPad Prism for Windows was used for statistical analysis. A *p* value < 0.05 was considered statistically significant.

## 3. Results

### 3.1. Irreversible Electroporation (IRE) in Combination with Intratumor Administration of c-di-GMP Adjuvant Has Therapeutic Effect in a Murine Model of Melanoma

IRE produces cellular destruction and the release of tumor-specific antigens, which might be captured by antigen presenting cells to initiate the induction of an antitumor immune responses. However, the tumor microenvironment is not favorable for antitumor immune priming. We proposed that utilizing an immunotherapeutic approach in combination with IRE might favor the induction of stronger antitumor immune responses. In order to do this, IRE was combined with the simultaneous injection of the immunostimulatory agent and STING agonist, c-di-GMP. To evaluate this combination therapy, we first used a murine model of melanoma based on the administration of B16.OVA tumor cells. Mice bearing B16.OVA were treated with (i) IRE, (ii) intratumoral injection of c-di-GMP, (iii) intratumoral injection of c-di-GMP immediately accompanied by IRE, or (iv) left untreated (control group).

**Figure 1:**
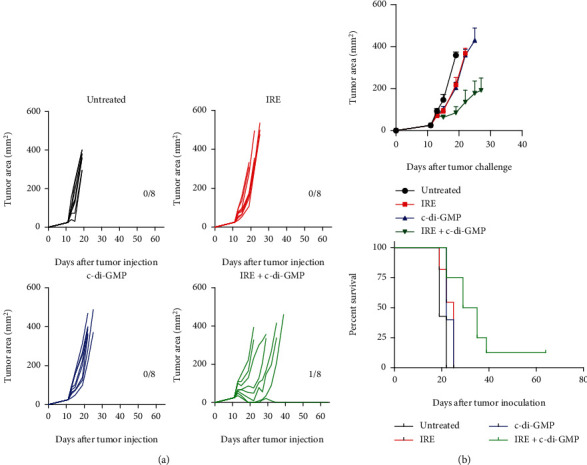
Treatment of B16.OVA tumor cells by irreversible electroporation plus c-di-GMP. Mice were challenged s.c. with B16-OVA tumor cells and at days 7-10, when tumors reached 5 mm in diameter, they were treated i.t. as indicated. (a) Each curve represents tumor mean diameter for an individual mouse. Numbers of mice free of tumors out of the total animals per group are indicated. (b) The Kaplan-Meier plots of the percentage of mice survival are represented. Log-rank (Mantel-Cox) test. *p* < 0.05.

IRE treatment or c-di-GMP treatment alone did not show any effect on tumor kinetics and did not significantly decreased tumor growth compared to the untreated group (Figures 1(a) and 1(b)). However, mice that received IRE + c-di-GMP combination treatment showed a significant delay in tumor growth, resulting in 1 out of 8 mice completely rejecting the tumor (Figure 1(a)). Survival was significantly improved in those mice compared to single treatment groups and the untreated control group (*p* < 0.05; Figure 1(b)).

### 3.2. IRE in Combination with c-di-GMP Has a Therapeutic Effect in a Murine Model for Hepatocellular Carcinoma

**Figure 2:**
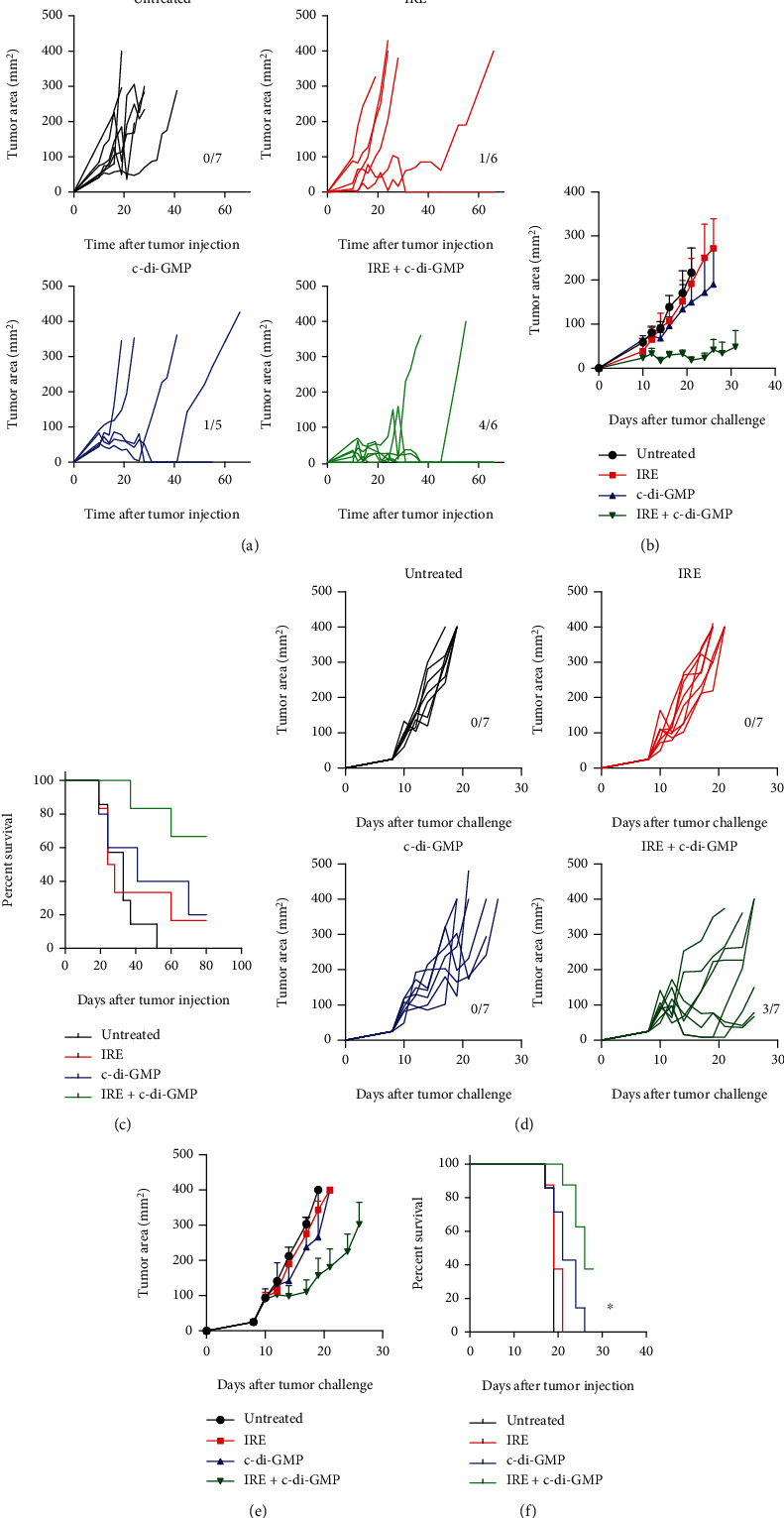
Treatment of PM299L tumor cells by irreversible electroporation plus c-di-GMP. Mice were challenged s.c. with PM299L tumor cells and at days 7-10, when tumors reached 5 mm in diameter, they were treated i.t. as indicated. (a) Each curve represents tumor mean diameter for an individual mouse. Numbers of mice free of tumors out of the total animals per group are indicated. (b) The Kaplan-Meier plots of the percentage of mice of survival are represented. Log-rank (Mantel-Cox) test, *p* < 0.05.

IRE is an emerging alternative to ablative therapies for liver cancer [1]. Even if results were particularly promising for small-size and very early-stage hepatocellular carcinomas (HCC), tumor recurrence is still high [24, 25]. We tested if combination of IRE with the c-di-GMP adjuvant could improve the efficacy of IRE in a murine model of HCC. C57BL/6 mice were injected with PM-299L hepatoma cells subcutaneously. Seven days later, mice were treated with (i) IRE, (ii) intratumoral injection of c-di-GMP, or (iii) intratumoral injection of c-di-GMP followed immediately by IRE or (iv) left untreated. It was observed that IRE treatment alone or c-di-GMP alone cured 16.6% and 20% of mice, respectively (Figure 2(a)). Surprisingly, 66.7% of mice responded to IRE + c-di-GMP combination therapy (Figure 2(a)) with 4 out of 6 mice totally rejecting established tumors. On the other hand, only 1 out of 5 or 1 out of 6 mice were cured after c-di-GMP or IRE monotherapies, respectively (*p* < 0.05; Figure 2(b)). We repeated the experiments but using male mice and the same treatments schedules. Combination therapy c-di-GMP plus IRE was also able to significantly delay tumor growth and mice survival (Figures 2(d)–2(f)), although the effect was less pronounced than that found in female mice. No effect was observed when mice were treated with monotherapies.

**Figure 3:**
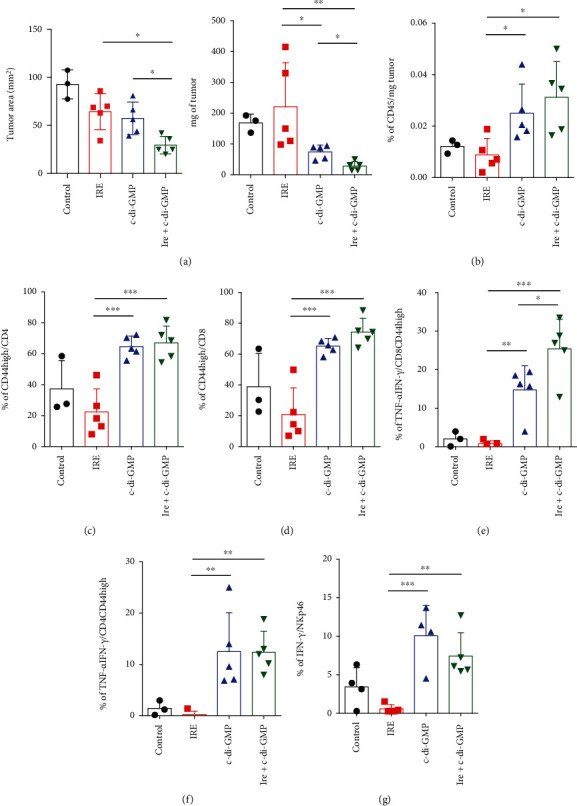
Phenotypic and functional analysis of intratumor T lymphocytes in mice bearing PM299L tumors. Mice were challenged with PM299L tumor cells s.c. and at days 7-10, when tumors reached 5 mm in diameter, they were treated i.t. as indicated and sacrificed seven days later for phenotypic analysis of tumor-infiltrating lymphocytes. (a) Tumor area (measured with a caliper) and tumor weight measured in each individual mice the day of sacrifice. (b–g) Phenotypic and functional analysis of tumor-infiltrating T lymphocytes and NK cells measured by flow cytometry using the indicated antibodies. One-way ANOVA with Tukey's multiple comparison test. *p* < 0.05; ∗∗*p* < 0.01; ∗∗∗*p* < 0.001.

In order to evaluate the antitumor immune response in vivo, we repeated the experiment with the same treatment options but sacrificing the mice ten days after tumor injection. The phenotype and functionality of tumor infiltrates was then analyzed. Tumor size at the day of sacrifice was significantly lower in mice treated with the combination therapy (both measured as tumor diameter and as tumor weight, Figure 3(a)). Flow cytometric analysis of tumor-infiltrating lymphocytes showed a significant increase in the number of leukocytes (percentage of CD45^+^ cells/mg of tumor) in mice treated with c-di-GMP alone or with c-di-GMP combined with IRE (Figure 3(b)). These differences were also observed in the percentage of activated CD4^+^ and CD8^+^ infiltrating lymphocytes (the percentage of CD44^high^CD4^+^ and CD44^high^CD8^+^ T cells) (Figure 3(c) and 3(d)). Importantly, these two groups showed a significant increase in the percentage of CD4^+^ and CD8^+^ T cells that simultaneously expressed TNF-*α* and IFN-*γ*, and in the percentage of IFN-*γ*-producing NK cells (Figures 3(e)–3(g) and Figure S1). These results suggest that intratumoral administration of c-di-GMP induced a proinflammatory microenvironment favorable for T cell/NK cell activation. IRE treatment alone did not induce tumor infiltration of immune cells. Interestingly, the combined therapy of c-di-GMP and IRE was able to significantly increase the percentage of infiltrating activated IFN-*γ* and TNF-*α*-producing CD8^+^ T cells, suggesting that this combination therapy favors the activation of an antitumor immune response able to control tumor growth more efficiently.

## 4. Discussion

IRE is a promising, low-invasive technique for the ablation of solid tumors. Unlike thermal ablation techniques, IRE treatment does not damage the surrounding extracellular matrix, vessels, nerves, and neighboring normal tissue [12, 13, 26, 27]. Clinical trials have shown safety and absence of serious adverse effects related to the procedure. However, the therapeutic efficacy is poor [5, 12, 13], and high incidence of short-term recurrences was reported [12, 28, 29]. Some studies suggest that the remaining islands of viable tumor cells within ablated regions after IRE treatment are responsible for higher resistance to pore formation [14]. It is probable that these remaining IRE resistant cells may continue tumor development and reduce the therapeutic efficacy of this technique.

Long-term tumor growth control can be achieved by eliciting a strong antitumor immune response. However, IRE alone does not induce favorable inflammatory conditions to facilitate antitumor T cell priming. As shown in this work, IRE treatment did not augment T cell infiltration of the tumor or improve infiltrating T cell activation state. IRE-induced cellular destruction may lead to the release of a substantial amount of tumor-specific antigens that can be engulfed by dendritic cells (DC, the professional antigen presenting cells) for their presentation to tumor-specific T lymphocytes. However, T lymphocyte activation is only achieved if DCs are in a mature stage. This maturation process is highly impaired by the immunosuppressive tumor microenvironment. Modifying the tumor microenvironment by introducing molecules that promote the maturation of dendritic cells might favor the activation of an antitumor immune response. We speculated that intratumoral injection of factors with proinflammatory properties, like c-di-GMP, might synergize with IRE technique to elicit antitumor T cell responses.

In previous work, we showed that the therapeutic effect of IRE can be improved when combined with simultaneous intratumoral administration of Poly-ICLC, a TLR3 agonist that mimics a viral infection and activates a strong innate immunity [16]. In addition to TLR ligands, the cGAS–STING axis was identified as an important regulator of immunity by mediating type I IFN production in response to cytosolic DNA [30, 31]. Type I IFN production elicited through the STING pathway has an essential role in the development of antitumor immunity by facilitating antigen cross-presentation by DCs (reviewed in [32]). DNA sensing by STING triggers the production of type I IFN by DCs and facilitates effective cross-priming of tumor-specific CD8^+^ T cells [33]. The proinflammatory potential of STING signaling has prompted many laboratories towards the search and development of small molecule modulators targeting the cGAS–STING–TBK1 signaling pathway for their clinical use as a new immune stimulatory therapy. While multiple new generation STING agonists are being advanced into clinical development (reviewed in [19]), data from initial phase I clinical trials showed that STING agonists alone elicited modest therapeutic efficacy [34]. This poor efficacy was in part due to their poor pharmacokinetic profile. The anionic properties of STING agonists reduce their membrane permeability, limiting their entry into the cytosol and the activation of the STING pathway. Moreover, systemic delivery of STING agonists for cancer therapy can induce off-target generalized inflammation or autoimmunity, since they do not preferentially localize to tumor tissue. We hypothesized that the anionic charge of the STING agonist c-di-GMP could facilitate its internalization into the tumor cells in vivo through the nanopores in the cell membrane caused by the IRE procedure, as it has been proposed by other means, such as the use of liposomal encapsulation [35]. Moreover, dead tumor cells loaded with STING agonists could be engulfed by DC and improve their maturation and the induction of a tumor-specific T cell immune response. In this scenario, we proposed the combination of intratumoral injection of c-di-GMP immediately followed by IRE as a more efficient antitumor therapy. We found that combination of IRE and c-di-GMP was able to delay tumor growth in two murine tumor models. We observed a significant delay in tumor growth B16.OVA melanoma and PM299L HCC tumor models. Interestingly, female mice responded more efficiently to combined therapy than male mice. This result is in agreement with previous reports showing that female mice respond better to immunotherapy [36]. Gender influence on cancer immunotherapy has been recently reviewed by Irelli et al. [37].

Image-guided locoregional therapies have increased substantially the overall 5-year survival of patients with liver cancers. However, new and more efficient treatment approaches are warranted to further improve treatment outcomes. The combination of local and systemic therapies is being actively studied to increase response rates (reviewed in [38]). Combination of locoregional therapies, such as local radiation, thermal ablations, or transarterial chemoembolization, with the systemic administration of immune checkpoint inhibitors has demonstrated increased antitumor immune response and constitutes a promising combination [39–41]. Intratumor administration of oncolytic viruses in combination with anti-PD1 antibodies is currently being investigated in clinical trials (NCT03071094, NCT02509507). Combination of novel immunotherapeutic strategies with locoregional therapies is indeed a treatment concept being actively developed. Several clinical trials have been initiated to test the combination of immune checkpoint blockade and other immunotherapies plus locoregional therapies (reviewed in [42]). All these trials will shed more light on the mechanisms of action of these combined therapies and will guide clinicians in designing more effective therapeutic strategies for each patient.

Our results show that the combination of IRE with STING agonist favors the activation of an antitumor T cell immune response compared to the single intratumoral administration of c-di-GMP or IRE treatment alone. This study have several limitations. New studies are needed to improve the efficacy of this combined therapy. An optimization of the IRE protocol, number of pulses and voltages, dose of STING agonist, delivery route, or repetitions of the therapy at different time points should be tested. In addition, a deeper analysis of immunological effects of locoregional therapies and synergies with immunomodulatory agents will help in the understanding of the mechanism of action of this combination therapy. Also, other variables such the age of animals, the type of tumors, or tumor heterogeneity, which may affect to immunotherapies [43, 44], should also be evaluated. Despite these limitations, and the difficulty of extrapolating preclinical data to clinical practice with patients, the present data could constitute the basis for clinically testing this combination therapy in refractory HCC.

## Data Availability

All data supporting the results has been included in the manuscript.

## Supplementary Materials

Supplementary Materials Figure S1: phenotypic and functional analysis of intratumor T lymphocytes in mice bearing PM299L tumors. Mice were challenged with PM299L tumor cells s.c., and at days 7-10, when tumors reached 5 mm in diameter, they were treated i.t. as indicated and sacrificed seven days later for phenotypic analysis of tumor-infiltrating lymphocytes. Phenotypic and functional analysis of tumor-infiltrating T lymphocytes measured by flow cytometry using the indicated antibodies. One-way ANOVA with Tukey's multiple comparison test, *p* < 0.05; ∗∗*p* < 0.01; ∗∗∗*p* < 0.001.
