# Supplementary figures and images for: Intratumoral STING Agonist Injection Combined with Irreversible Electroporation Delays Tumor Growth in a Model of Hepatocarcinoma
Source: Biomed Res Int. 2021 Jan 27;2021:8852233. doi: 10.1155/2021/8852233 (PMC7857890; doi:10.1155/2021/8852233)

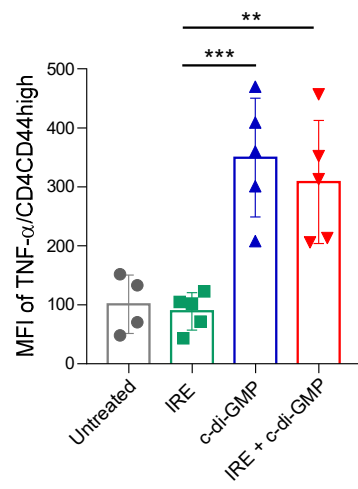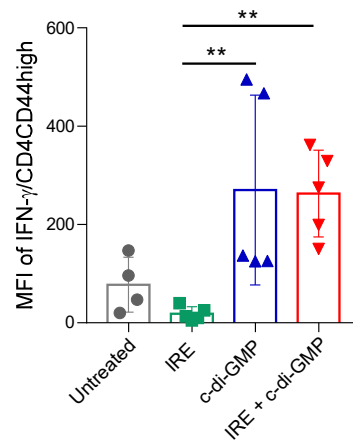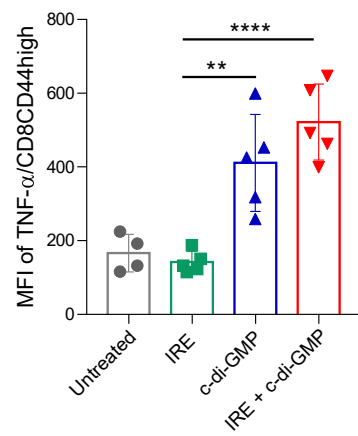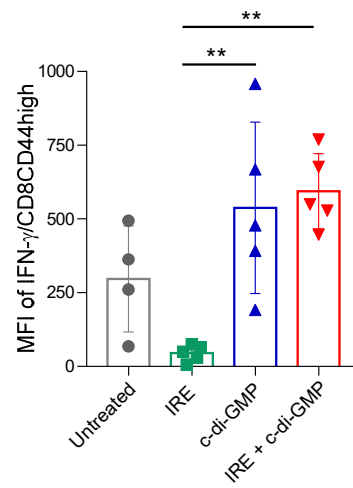

Supplement: Supplementary Materials — Figure S1: phenotypic and functional analysis of intratumor T lymphocytes in mice bearing PM299L tumors. Mice were challenged with PM299L tumor cells s.c., and at days 7-10, when tumors reached 5 mm in diameter, they were treated i.t. as indicated and sacrificed seven days later for phenotypic analysis of tumor-infiltrating lymphocytes. Phenotypic and functional analysis of tumor-infiltrating T lymphocytes measured by flow cytometry using the indicated antibodies. One-way ANOVA with Tukey's multiple comparison test, p < 0.05; ∗∗p < 0.01; ∗∗∗p < 0.001. [file 8852233.f1.pdf]
